# Evaluation of the effectiveness of a WHO-5A model based comprehensive tobacco control program among migrant workers in Guangdong, China: a pilot study

**DOI:** 10.1186/s12889-018-5182-6

**Published:** 2018-02-27

**Authors:** Wenxin Chai, Guanyang Zou, Jingrong Shi, Wen Chen, Xiao Gong, Xiaolin Wei, Li Ling

**Affiliations:** 1 Faculty of Medical Statistics and Epidemiology, School of Public Health, Sun Yat-sen University, Guangzhou, People’s Republic of China; 2 Sun Yat-sen Center for Migrant Health Policy, Sun Yat-sen University, Guangzhou, People’s Republic of China; 3 Institute for Global Health and Development, Queen Margaret University, Edinburgh, UK; 4 Division of Clinical Public Health and Institute for Health Policy Management and Evaluation, Dalla Lana School of Public Health, University of Toronto, Toronto, Canada

**Keywords:** Cotinine, Tobacco control, Intervention programs, Migrant, Workplace

## Abstract

**Background:**

As a vulnerable population in China, migrant workers have a higher smoking rate than the general population. This study aims to assess the effectiveness of a WHO-5A based comprehensive tobacco control program in workplaces aggregated with migrants.

**Methods:**

Using a controlled before and after design, four purposely selected manufacturing factories were assigned to either intervention or control groups. Participants in the intervention arm received adapted 5A group counseling regularly supported by social-media and traditional health education approaches. The primary outcome was the change of smoking rate based on salivary cotinine concentration at three-month follow-up as compared to the control arm. Secondary outcomes were changes in smoking-related knowledge and attitudes assessed using questionnaires. Difference-in-differences approach (DID) and generalized estimating equations (GEE) models were used to conduct the effectiveness analysis.

**Results:**

149 and 166 workers were enrolled in the intervention and control arm respectively. The multiple imputed and adjusted GEE models demonstrated that, compared to those in the control arm, participants in the intervention arm had nearly 2.4 times odds of improving smoking-related knowledge (OR = 2.40, 95% CI = 1.32–4.36, *P* = 0.02) and three times the odds of improving smoking-related attitude (OR = 3.07, 95% CI = 1.28–7.41, *P* = 0.03). However, no significant difference was found regarding the change of smoking rate between the two arms (*P* > 0.05). The regression analysis showed that attendance at the 5A group counseling sections was an important determinant of stopping smoking or improving smoking-related knowledge and attitudes in the intervention group.

**Conclusions:**

This WHO-5A comprehensive intervention was effective in improving migrant workers’ knowledge of smoking and anti-smoking attitudes. A large-scale, long-term trial is recommended to determine the effectiveness of this intervention.

**Trial registration:**

ChiCTR-OPC-17011637 at Chinese Clinical Trial Registry. Retrospectively registered on 12th June 2017.

**Electronic supplementary material:**

The online version of this article (10.1186/s12889-018-5182-6) contains supplementary material, which is available to authorized users.

## Background

Tobacco use is the second leading global risk for mortality after high blood pressure and contributes to 9% of premature deaths such as heart disease, diabetes and cancers in the world [1]. Among the global smoking population, about one third are from China, where the smoking rate was up to 27.7% (52.9% for men and 2.4% for women) [2]. To date, no comprehensive law on tobacco control is available at the national level [3]. Several laws and regulations are available at the local level, although the progress and effect on tobacco control varied across geographical areas. It was not until the end of 2014 that the Cigarette Control Regulations in the Public Areas, including workplaces, was drafted and reported to the State Council of China, but it has not yet been issued nationwide [4]. Data from the International Tobacco Control (ITC) project shows that China has the highest rate (70%) of smoking in the workplaces in the world [5].

China has been experiencing unprecedented internal migration since the reform and opening. In 2014, the migrant population was up to 253 million. Migrants contribute to the major workforce in China. About 75% of migrants were employed in the manufacturing, construction, wholesale and retail trade, hotels and catering services and other tertiary services [6]. Studies showed that the smoking rate among migrant workers was 32.5% (55.3% for male and 1.9% for female) [7], higher than that of the general population [2]. Many socioeconomic factors such as poor education [6], work pressure [8], as well as migration-related features [8, 9] such as the numbers of migratory cities and the duration of stay, contributed to the high smoking prevalence.

However, the high smoking rate was not a unique problem among Chinese internal migrants, but an international problem [10–12]. For instance, a larger scale study in Europe found that migrants were more likely to smoke than non-migrants [10]. Another study revealed that the smoking rate in an Indian city was up to 90% among interstate migrant construction workers [11].

Smoking cessation in any population and at any age reduces smoking-related mortality [13]. Conducting smoking cessation interventions in the workplaces, including recruitment and regular follow-ups is necessary, given the high smoking rate among the workers; but also feasible as they work together and spend most of their daily time in their workplace [14]. Previous studies have suggested the efficacy of smoking cessation interventions in the workplace [15–19]. Several intervention approaches have shown to be effective for tobacco control including individual or group counselling, pharmacotherapy, and incentives. However, these approaches are often used in developed countries [15–17]. In developing countries, approaches such as environmental support or self-help interventions were often reported, which were however shown to be less effective [18, 19]. More importantly, tobacco control intervention among the migrant workers is rarely reported.

The 5A model [20], based on a systematic approach of “Ask, Advice, Assess, Assist, Arrange” is recommended for planning smoking cessation programs by the WHO [21]. This model has shown to be an effective way to help patients to quit smoking in primary care outpatient settings in most of the developed countries [22, 23]. To our knowledge, few studies have reported the use of WHO-5A model in developing countries [24]. It has not yet been used in non-medical workplaces and its application and effectiveness among Chinese migrant workers are unknown.

Hence, we developed a comprehensive intervention program based on WHO-5A model to reduce smoking in the workplace. In this paper, we aim to understand the feasibility and effectiveness of this intervention and examine the factors influencing the effectiveness of the intervention.

## Methods

### Settings and participants

This study was conducted in Zhongshan City, Guangdong Province. Guangdong has the largest migrant worker population in China [7]. Located in the Pearl River Delta Areas of Guangdong, Zhongshan is a major city aggregated with manufacturing factories mostly staffed with migrant workers.

Four manufacturing factories were selected from the list of factories provided by the government and were allocated to intervention and control groups according to the willingness to cooperate of these factories. Inclusion criteria for the cluster recruitment were: 1) labor-intensive manufacturing factories; 2) the managers should be willing to adhere to the planned intervention. We excluded factories that had taken part in any tobacco control interventions before. In each selected factory, we recruited migrant workers who were ≥16 years old and willing to participate in the study. We excluded migrant workers who had difficulty in understanding and completing the questionnaire even with the help of facilitators.

We estimated that the intervention would achieve a reduction of the primary outcome (smoking rate). According to the previous study [8], the smoking rate among migrant workers in China was 32%. The sample size was estimated based on the assumption of 5% significant level, 80% power and the expected smoking rate of 20% [25]. Assuming a 50% attrition rate and a within-subject correlation coefficient of 0.1, the sample size for each arm was calculated to be 115 smokers.

### Intervention

**Fig. 1:**
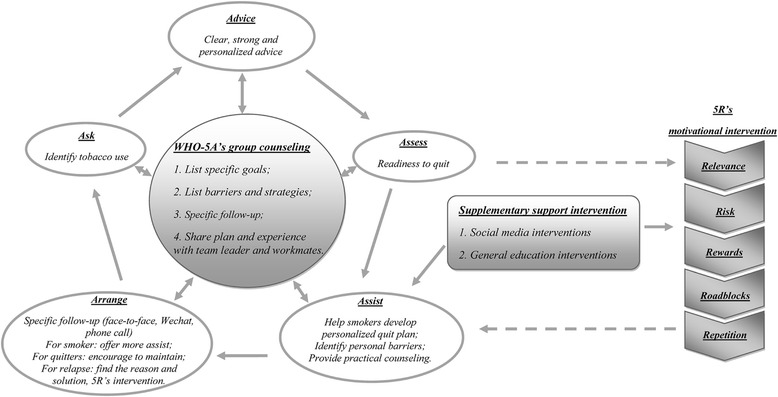
The adapted WHO-5A group counseling cycle diagram

The two intervention factories were provided with the WHO-5A-based comprehensive smoking cessation intervention, while the control factories were not provided any new intervention. The key components of the intervention are provided in the WHO-5A group counseling cycle diagram (Fig. 1).

**Fig. 2:**
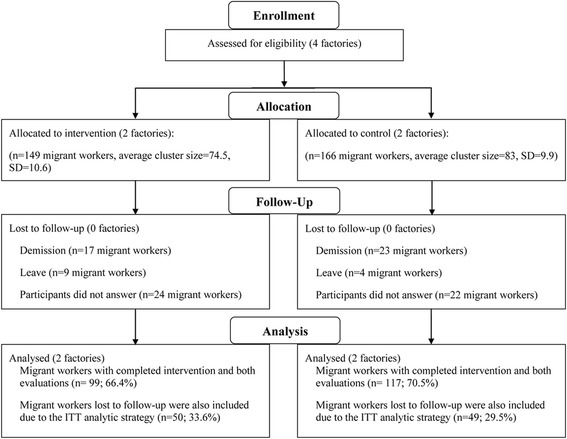
Flow diagram of progress of clusters and individuals

### WHO-5A group counseling package

A group counseling package including an operational guideline and a training module was developed based on the WHO-5A tobacco cessation model [21]. This package was designed to be brief, pragmatic and comprehensible considering the characteristics of migrant workers, such as poor educational levels, low income, high mobility, and intensive workload.

In each intervention factory, a team leader was selected from every six to eight migrant workers and invited to participate in a training workshop delivered by the researchers. In the workshop, researchers introduced the details of this study to the selected team leaders and trained them how to deliver group counseling to their team members based on the operational guideline. In addition, the researchers trained the team leaders to make plans for quitting smoking and follow-up. The workshop lasted 1.5–2 h.

Based on the WHO-5As group counseling (Ask, Advise, Assess, Assist, Arrange), specifically, the team leaders Asked members’ current smoking status, Advised smokers to quit in a clear, strong and personalized manner. The team leaders educated their members on the acute, long-term and environmental risks of smoking, demonstrated that smoking can worsen their existing health problems, increase economic burden of their family, and threaten the work safety. Members were also informed of the potential rewards of smoking cessation such as improved health and self-image, being more popular, and saving money to support the family. Given the poor knowledge of tobacco hazards in migrant workers, the team leaders used supplementary materials developed by the research team to help enhance members’ quitting motivation, if necessary.

Then, smokers were Assessed for the readiness of quitting. For those willing to quit, the team leaders would Assist the participants with individual quitting plans, specialist cessation methods (i.e. sports, reading, chatting, free gum offered by the research team). The frequency of Arrangement and follow-ups was flexible and up to the members’ availability and quitting process, but at least once a week. Supplementary cessation assistance such as social media and self-help materials were provided, which are described in detail in the following paragraph. For those not willing to quit, the team leaders delivered a 5Rs (Relevance, Risks, Rewards, Roadblocks, Repeated) motivational counseling intervention in every group counseling, until they were ready to quit. Every group counseling session lasted for 10 to 15 min.

### Supplementary interventions

#### Social media interventions

A social media called WeChat (Tencent Ltd., Shenzhen, China) and telephones were employed to supplement smoking cessation intervention in the intervention arm. WeChat is the most popular messaging programme used by people of all ages and professions in China [26]. A multifunctional background interface in WeChat was used to deliver tobacco-related text, picture or video messages to help smokers to quit every 2 weeks. A real-time chat room in WeChat was also set up for participants in each intervention factory respectively. Participants in real-time chat rooms could seek assistance from the team leaders, and share their experiences of smoking cessation with the other participants. Team leaders conducted tobacco-related counseling for participants who had no WeChat through telephone once a week.

#### General health education

Two rounds of smoking-related open lectures were held for the participants in the intervention arms to reinforce the WHO-5A group consulting intervention by the research team. Self-help materials including six-page leaflets on tobacco control and a book called ‘*The easy way to stop smoking*’ were distributed to every participant. A series of educational posters were also displayed in the intervened factories.

Quality control activities including weekly recording forms and field visits were conducted to improve the fidelity of intervention every 2 weeks.

The intervention was implemented for 3 months from October to December 2015, and the follow-up data collection was in January 2016. A flowchart of the allocation, intervention and follow up is shown in Fig. 2.

### Outcomes

***The primary outcome*** was the change of smoking rate among migrant workers, based on the salivary cotinine concentrate. Cotinine is the major primary metabolite of nicotine in the human body and can be used as a biomarker of recent smoking status in population studies [27]. In this study, we collected saliva samples rather than blood and urine samples as saliva samples were noninvasive and easier to collect. A cutoff of 15 ng/mL of salivary cotinine concentrate was used to distinguish active smokers from nonsmokers [26]. All participants provided at least 2 ml saliva samples for the salivary cotinine concentrate test. We required all participants to refrain from eating, drinking, or smoking at least 1 h before sample collection. We collected salivary samples on the day of transportation, and samples were labeled and stored in EP tubes (Eppendorf Tubes) at − 20 °C until laboratory analysis [27]. Samples were analyzed using the liquid chromatography-tandem mass spectrometry (LC/MS/MS) method. All experiments were duplicated. The laboratory technicians were blind to participants’ smoking status.

***The secondary outcomes*** were changes in tobacco-related knowledge and attitudes.

Participants completed self-administered questionnaires including demographic characteristics and migration status, employment status, smoking behaviors, smoking-related knowledge and attitudes towards smoking at baseline and at the end of three-month follow-up. Smoking-related knowledge and attitudes were measured by thirteen items and fifteen items, respectively (Table 1). The degrees in each item were measured by two scales: 0 (don’t know) and 1 (know) for knowledge; 0 (negative or neutral), 1 (positive) for attitudes. The total knowledge and attitudes scores ranged from 0 to 13 points and 0–15 points, respectively: the higher scores, the better knowledge of tobacco and the stronger anti-smoking attitudes.

**Table 1:** Outcome variables and items of the intervention effects

Outcome variables	Items
Smoking status	Smoking status was based on the salivary cotinine concentrate.
Smoking-related knowledge	
Tobacco-related diseases	Lung cancer
	Emphysema
	Accelerated aging
	Cardiopathy
	Oral cancer
	Abortion
	Impotence
	Chronic bronchitis
	Stroke
SHS	Do you think second-hand smoking was harmful
SHS-related diseases	Lung cancer
	Other respiratory system diseases
	Cardiovascular diseases
Attitudes towards smoking	
	Everyone has got to die of something, so why not enjoy yourself and smoke.
	People could easily get addicted to smoking.
	Cigarette companies should not be allowed to advertise by sponsoring athletic events.
	Smoking is a waste of money.
	Smoking enhances popularity and social bonding.
	Low tar cigarettes are less harmful than regular cigarettes.
	Smoking is a sign of maturity.
	Tobacco companies do good things for Chinese society.
	The government should do more to control smoking.
	The way a smoker inhales can affect the amount of tar and nicotine a smoker takes in.
	Filters reduce the harmfulness of cigarettes.
	Smoking helps you relieve your stress.
	Smoking helps you refresh yourself and relieve your fatigue.
	Discouragement from families is an important reason to quit smoking.
	It is not difficult to quit smoking if you want to do so.

The study was approved by the Institute Review Board of School of Public Health, Sun Yat-sen University (Ref. 44/2015). All participants were informed of the details of this study and provided written consent. The study is retrospectively registered with the Chinese Clinical Trial Registry in June 2017 (ChiCTR-OPC-17011637), based on the WHO’s clinical trial definition [28]. The overall study protocol and CONSORT checklist are available as Additional files 1 and 2. This study was conducted in accordance with the study protocol.

### Statistical analysis

The data were double entered using Epidata 3.1. IBM SPSS Statistics 21 was employed for analysis. Descriptive analyses were used to describe migrant characteristics; means and standard deviations were used for continuous variables, whilst counts and proportions for categorical variables. Independent *t*-tests and chi-square test were used to compare the difference of baseline characteristics between the intervention and control arms. To evaluate the effects of the intervention, we performed a difference-in-difference analysis. For study group *i* at time period *t*, we used generalized estimating equation (GEE) models to estimate the following:

$$ g\left\{\mathrm{E}\left(\mathrm{Yit}\right)\right\}={\beta}_0+{\beta}_1\ast {Group}_{it}+{\beta}_2\ast {Time}_{it}+{\beta}_3\ast {Group}_{it}\ast {Time}_{it}+{\beta}_4\ast \operatorname{cov}\mathrm{ariates}+{\varepsilon}_{it}, $$

where g{} is the link function; *Y* is the outcomes, either the smoking status, smoking-related knowledge score, or smoking-related attitude score; *Group*_*it*_, where the control group was 0, and the intervention group was 1; *Time*_*it*_, where baseline was 0, and follow-up was 1; The difference-in-differences term, *Group*_*i*_ *Time_*t*_, was our focal independent variable, defined as the interaction of a pre-post intervention difference between two study groups; The *β*_3_ coefficient for the difference-in-difference term is the main parameter of interest and intended to estimate the effect of the intervention on the outcomes; *ε*_*it*_ was the residual error. We used a logit link for the binary outcome and an identity link for the scale outcomes as link functions, and the working correlation matrix was selected to be exchangeable. A crude and baseline covariate adjusted GEE models were performed, to obtain *odds ratio* (OR) with 95% confidence intervals (95% CI) to assess the effects of the intervention.

Analyses followed the intention-to-treat (ITT) principle, and all migrant workers that were recruited and provided data at baseline were included in the effectiveness analysis of this intervention. To examine the implications of missing values and sample attrition for the study conclusions, we used multiple imputations (MI) to address missing or incomplete data in addition to available case analysis [29], and the results were based on the analysis of both the available case datasets (excluding all subjects with missing data) and MI datasets (including all subjects who participated in the baseline survey). The MI analyses were based on 50 data sets with missing values imputed via multiple multivariate regressions by chained equations, and the results were combined from the 50 imputed data sets based on Rubin’s rule. The imputation models included the study group indicator, time indicator, outcome measures at baseline and follow-ups and baseline covariate described in Table 2. Logistic regressions and multiple linear regressions were respectively employed to explore factors associated with the change of the outcome variables from baseline to three-month follow-up in the intervention arm. A 5% *p*-value (*p* < 0.05) will be used to indicate statistical significance and all tests were 2-sides.

**Table 2:** Baseline comparison of intervention and control arms

Variables	Intervention arm	Control arm	Variables	Intervention arm	Control arm
Demographic characteristics	149(47.30)	166(52.70)	Migration status		
Gender*, n(%)			Migration with family, n(%)		
Male	136(91.28)	137(82.53)	Yes	115(77.18)	119(71.69)
Female	13(8.72)	29(17.47)	No	34(22.82)	47(28.31)
Age**, n(%)			Migratory times, n(%)		
≤ 25	7(4.70)	22(13.25)	1	24(16.11)	24(14.46)
26–35	38(25.50)	59(35.54)	2–3	93(62.42)	110(66.47)
≥ 36	104(69.80)	85(51.21)	≥ 4	32(21.48)	32(19.27)
Marital status, n(%)			Migratory duration, n(%)		
Unmarried	31(20.81)	50(30.12)	≤ 5	18(12.08)	39(23.49)
Married	118(79.19)	116(69.88)	6–10	32(21.48)	35(21.08)
Education status, n(%)			11–15	41(27.52)	43(25.90)
Primary or below	31(20.81)	25(15.06)	≥ 16	58(38.93)	49(29.52)
Junior	83(55.70)	91(54.82)	Health status		
Senior	28(18.79)	42(25.30)	Reported having physiological illness, n(%)	61(40.94)	80(48.19)
College or higher	5(3.36)	8(4.82)	Smoking-related variables at baseline		
Income (Yuan)**, n(%)			Smoke **, n(%)	83(55.70)	58(34.94)
≤ 2000	13(8.72)	45(27.11)	Average daily cigarette consumption**, mean ± SD	6.61 ± 8.39	3.35 ± 5.86
2001–3000	77(51.68)	84(50.60)	Average monthly costs on smoking (RMB), mean ± SD	215.21 ± 149.78	198.98 ± 137.19
3001–4000	49(32.89)	25(15.06)	Confidence of success in smoking cessation(1–5), mean ± SD	2.94 ± 1.23	3.12 ± 1.25
≥ 4001	10(6.71)	12(7.23)			
Employment status					
Attendance time, n(%)					
Day	140(93.96)	153(92.17)			
Night or double-shift	9(6.04)	13(7.83)			
Work-hours per day, mean ± SD	9.24 ± 1.41	8.93 ± 1.26			
Work place, n(%)					
Indoors	118(79.20)	139(83.73)			
Outdoors	11(7.38)	7(4.22)			
Both	20(13.42)	20(12.05)			

** chi-square test *P* < 0.001; *: chi-square test *P* < 0.05

## Results

### Baseline characteristics and overview of the intervention

In total, 149 and 166 participants were recruited from the intervention and control arm respectively. As compared to the control arm, the intervention arm had a higher proportion of participants who were male (91.28% vs. 82.53%, *P* < 0.05), aged over 36 years old (69.80% vs. 51.21%, *P* < 0.001), had a monthly income of 2000–4000 Yuan RMB (84.57% vs. 65.66%, *P* < 0.001). At the baseline, the smoking rate based on the salivary cotinine was higher in the intervention arm than the control arm (55.70% vs. 34.94%, *P* < 0.001). The average daily cigarette consumption was also higher in the intervention arm (6.61 ± 8.39 vs. 3.35 ± 5.86, *P* < 0.001). No significant difference was found between the two arms regarding marital status, education level, employment status, migration status, health status and average monthly costs of smoking (*P* > 0.05) (Table 2).

### Process of intervention implementation

Of the 149 and 166 participants, 3 months into intervention 33.6% (*n* = 50) and 29.5% (*n* = 49) were lost to follow up respectively in the intervention and control arm. Overall, 77.11% smokers (*n* = 64) attended the 5A group counseling in the intervention arm. On average, each smoker attended 7.67(±8.94) counseling sessions. Of all the smokers, 30.12% (*n* = 25) received smoking-related information from Wechat multifunctional background interface and 44.58% (*n* = 37) from the participants’ Wechat real-time rooms. 32.89% (*n* = 49) smokers attended the open lectures delivered by the research team and 71.81% (*n* = 107) noticed the posters (Table 3).

**Table 3:** Process of implementing the comprehensive smoking cessation intervention

Tobacco control efforts (*N* = 83)	n(%)/ mean ± SD
Participants attendingWHO-5A group counseling	
Attending 5A group counseling	64(77.11)
Times of attending 5A group counseling	7.67 ± 8.94
Participants attending supplementary support interventions	
Social media interventions	
Receiving smoking-related information from Wechat multifunctional background interface	25(30.12)
Receiving smoking-related information from the participants’ Wechat real-time rooms	37(44.58)
General health education	
Attending open lectures	49(32.89)
Reading the posters(*N* = 149)	107(71.81)

### Intervention effects

Table 4 shows the active smoking rates based on saliva samples, the scores of smoking-related knowledge and attitude at baseline and three-month follow-up in the intervention and control arm. We observed the increase of the smoking-related knowledge scores (4.37 ± 2.28 at baseline vs. 5.30 ± 2.82 in the available datasets and 5.47 ± 2.77 in the MI datasets at 3-month follow-up). We also observed the increase of smoking-related attitude scores (7.03 ± 3.27 at baseline vs. 8.21 ± 3.82 in the available datasets and 8.27 ± 3.67 in the MI datasets at three-month follow-up) in the intervention arm. In the control arm, similar scores were observed across assessment times regarding the smoking-related knowledge (5.04 ± 2.81 at baseline vs. 5.18 ± 2.72 in the available datasets and 5.28 ± 2.74 in the MI datasets at three-month follow-up) and attitude scores (8.08 ± 3.64 at baseline vs. 8.22 ± 3.70 in the available datasets and 8.22 ± 3.65 in the MI datasets at three-month follow-up). However, the cotinine tests of saliva samples did not show changes regarding the active smoking rates in the two arms.

**Table 4:** Smoking rates, and smoking-related knowledge and attitude scores at baseline and 3-month follow-up in the intervention and control arm

Outcomes		Baseline	Follow-up	
			Available case analysis	Multiple imputation analysis
Smoking rates, n(%)	Intervention	83(55.70)	55(55.00)	81(54.36)
	Control	58(34.94)	41(35.34)	56(33.73)
Smoking-related knowledge scores, mean ± SE	Intervention	4.37 ± 2.28	5.30 ± 2.82	5.47 ± 2.77
	Control	5.04 ± 2.81	5.18 ± 2.72	5.28 ± 2.74
Smoking-related attitude scores, mean ± SE	Intervention	7.03 ± 3.27	8.21 ± 3.82	8.27 ± 3.67
	Control	8.08 ± 3.64	8.22 ± 3.70	8.22 ± 3.65

Over the period from baseline to three-month follow-up, in comparison with participants in the control arm, participants in the intervention arm had 2.3–2.4 times more odds (available case analyses: OR = 2.33, 95% CI = 1.17–4.64, *P* = 0.02; multiple imputation analyses: OR = 2.40, 95% CI = 1.32–4.36, *P* = 0.02) of improving their smoking-related knowledge; participants in the intervention arm had nearly three times more odds (available case analyses: OR = 2.84, 95% CI = 1.04–7.77, *P* = 0.04; multiple imputation analyses: OR = 3.07, 95% CI = 1.28–7.41, *P* = 0.03) of improving their smoking-related attitudes. However, the GEE analysis showed that there were no significant differences in smoking rate changes based on saliva samples between the two arms (*P* > 0.05). In the adjusted GEE regression models, the results were consistent with the crude analyses (Table 5).

**Table 5:** GEE^a^ analyses of intervention effects on smoking rates, smoking-related knowledge and attitude

Outcomes	Available case analyses				Multiple imputation analyses			
	Crude analyses ^b^		Adjusted analyses ^c^		Crude analyses ^b^		Adjusted analyses ^c^	
	OR(95%CI)	p	OR(95%CI)	p	OR(95%CI)	p	OR(95%CI)	p
Smoking rates	0.98 (0.67–1.47)	0.95	0.98(0.66–1.47)	0.94	0.99 (0.70–1.41)	0.96	0.99(0.70–1.41)	0.96
Smoking-related knowledge scores	2.33(1.17–4.64)	0.02	2.31(1.16–4.60)	0.02	2.40(1.32–4.36)	0.02	2.41(1.33–4.38)	0.02
Smoking-related attitude scores	2.84 (1.04–7.77)	0.04	2.85 (1.04–7.80)	0.04	3.07(1.28–7.41)	0.03	3.08(1.28–7.44)	0.03

*OR* odds ratio, *CI* confidence interval

^a^Generalized estimation equation models to predict smoking abstinence taking into account within-subject correlation across 3 month follow-up

^b^Models included the predictor variables study group and time

^c^Models included the predictor variables study group, time, age, and monthly income

### Predictors of changes in smoking behavior, smoking-related knowledge, and attitudes

The binary logistic regression analysis results showed that the more sections of WHO-5A group counseling attended (Beta = − 0.059, OR = 0.943, 95% CI: 0.890–0.990) and the more confident the migrant smokers felt in achieving successful smoking cessation (Beta = − 1.664, OR = 0.189, 95% CI: 0.038–0.938), the more likely the participants were to change their smoking status into non-smokers based on saliva samples. Moreover, migrant smokers with health problems (Beta = 1.326, OR = 3.766, 95% CI: 1.187–11.950) tended to remain smoking (Table 6).

**Table 6:** Regression models of changes of smokers’ smoking status, smoking-related knowledge and attitudes scores in the intervention arm

Variables	*B*	*SE*	*Wald/t*	*P*	95%*CI*	
					Lower bound	Upper bound
Model 1. Logistic regression model of changes of smoker’ smoking status						
(Constant)	2.197	0.770	8.1441	0.004		
Times of attending WHO-5A group counseling	−0.059	0.029	3.9741	0.046	0.890	0.990
Confidence in achieving successful smoking cessation	−1.664	0.817	4.1541	0.042	0.038	0.938
Healthy problems	1.326	0.589	5.0661	0.024	1.187	11.950
Model 2. Multiple linear regression model of changes of smoking-related knowledge scores						
(Constant)	3.774	.758	4.978	< 0.001	2.288	5.260
Knowledge scores at baseline	0.419	0.115	3.650	< 0.001	0.194	0.664
WHO-5A group counseling	0.996	0.505	1.971	0.049	0.006	1.985
Educational posters	1.925	0.754	2.551	0.011	0.446	3.403
Migrant times						
1	Ref.					
2–3	−0.937	0.623	−1.505	0.132	−2.158	0.283
≥ 4	−2.062	0.731	−2.822	0.005	−3.495	−0.630
Model 3. Multiple linear regression model of changes of smoking-related attitudes scores						
(Constant)	7.770	1.147	6.776	< 0.001	5.523	10.017
Attitude scores at baseline	0.386	0.114	3.400	0.001	0.164	0.609
Migrant times						
1	Ref.					
2–3	−1.667	0.877	−1.900	0.057	−3.386	0.053
≥ 4	−3.240	1.047	−3.095	0.002	−5.291	−1.188

The multiple linear regression analysis shows that, after controlling for the baseline knowledge scores in the intervention arm, participants who attended the WHO-5A group counseling (Beta = 0.996, 95% CI: 0.006–1.985) and read the educational posters (Beta = 1.925, 95% CI: 0.446–3.403) tended to report higher knowledge scores. Participants who migrated for more times (Beta = − 2.062, 95% CI: -3.495-0.630 for at least four times migration, and Beta = − 0.937, 95% CI: -2.158-0.283 for two to three times migration) tended to report lower knowledge scores (Table 6). As for attitude, after controlling for the baseline scores in the intervention arm, participants who migrated for more times (Beta = − 3.240, 95% CI: -5.291 ~ − 1.188 for at least four times migration, and Beta = − 1.667, 95% CI: -3.386 ~ − 0.053 for two to three times migration) also tended to report lower attitude scores (Table 6).

## Discussion

Tobacco control is a key component of reducing non-communicable disease mortality [30]. Great global efforts geared towards smoking cessation have been made, such as the WHO Framework Convention on Tobacco Control approved in 2003, and many countries have made a series of strict tobacco control policies [30–32]. Although the global smoking problems have been improved, tobacco control among migrants all around the world is still relatively hard to conduct for various reasons, such as high mobility, high smoking rates, limited education levels, and psychological pressures [10]. Evidence on promoting smoking cessation practices among migrant populations is urgently needed.

To our knowledge, this is the first intervention study reporting the effectiveness of the WHO-5A-based comprehensive smoking cessation intervention in non-medical migrants-aggregated workplaces in China. Our study suggests that this comprehensive intervention program supported by social-media and general health education approaches was effective in improving smoking-related knowledge (OR = 2.40, 95% CI = 1.32–4.36, *P* = 0.02) and attitudes (OR = 3.07, 95% CI = 1.28–7.41, *P* = 0.03) among migrant workers as compared to control arms. The intervention program failed to reduce smoking rates based on the salivary cotinine concentration in the intervention arm.

### Smoking-related knowledge and attitudes

Existing studies have suggested that knowledge and attitudes play an important role in the initiation and maintenance of smoking cessation [33]. Our intervention was effective in improving participants’ smoking-related knowledge and attitudes. Smoking knowledge and attitudes scores were often used as parameters of intervention effectiveness. However, most of these studies occurred in schools or primary care settings rather than in the workplace [34–36]. Similarly, some school-based intervention studies also found significant effects on changing both smoking-related knowledge and attitudes [34, 35]. Other school-based intervention studies reported [36] that despite smoking knowledge increasing substantially over the follow-up, the attitudes did not change, mainly because attitudes were more stable than knowledge and more resistant to change.

Our study finds that increasing frequency of migration was a risk factor for migrant workers to change their knowledge and attitudes about smoking. Frequent migration indicated unstable living and working conditions, associated with life pressure [37]. Participants with more frequent migration might continue to smoke to cope with the life pressure with poorer receptiveness to the healthy knowledge about smoking and change the attitudes. Attending the 5A group counseling and reading the posters also helped to improve smoking-related knowledge and attitudes. Previous studies suggested the importance of 5A intervention and general education in improving knowledge and attitudes [15, 16]. We did not find other smoking cessation efforts such as social media interventions or open lectures as being important predictors, perhaps due to the limited coverage (Table 3).

### Smoking rate

In this study, we did not find a significant change in smoking rates in the intervention group as compared to the control group over 3 months. This was not surprising - another short-term study, Okechukwu’s four-month multi-pronged intervention, did not find a significant effect on smoking cessation at the end of the intervention [38]. A significant smoking reduction was reported in longer-term interventions, for example, Bergstrom’s three-year comprehensive workplace health promotion program [39], and in Takashi’s 10-year intervention program in Japanese male workers [40]. Indeed, 3 months was not long enough to completely stop smoking or sustain the effect of an intervention. In addition, the small sample size and high loss-to-follow-up rate may also have led to the insignificant difference in the change of smoking status between the two arms.

Consistent with other studies [41, 42], our study found that migrants attending 5A group counseling more frequently tended to quit smoking. Like the studies in East Asian countries such as Japan, Thailand, and Malaysia [43, 44], we found that greater confidence of success in smoking cessation also helps to stop smoking. However, some studies from the developed countries suggested vice versa [45]. The inconsistent finding could be plausibly due to the cultural differences or a more recent history of tobacco control efforts in Asia [46]. Our results suggest that smokers without physiological illnesses were more likely to quit. A possible explanation was that healthy smokers were concerned more about their health and so were more likely to develop healthier lifestyles.

### Strengths

Our study suggests that the intervention was feasible in migrant-aggravated workplaces. We tailored the WHO-5A protocol into a 5A group guideline and training module [21] and used it to train the team leaders in a manufacturing context. These 5A group guidelines and training modules were operational and feasible for team leaders to conduct appropriate interventions in migrant-aggravated manufacturing settings. The team leader approach allowed the intervention delivery to be well embedded in the routine and busy manufacturing work, which allowed migrant workers to receive personalized advice. We delivered tobacco-related texts and multimedia content to the migrant workers through popular social media such as WeChat. The heavy workload made it difficult to provide adequate group consulting services to participants. However, social media provides an opportunity for migrant workers to receive timely educational messages and interact with other workmates on smoking cessation experiences. Considering the poorer educational and social economic status of migrants, we tailored and simplified the intervention messages to enable the achievement of significant improvement in knowledge and attitude. Smoking status was often assessed based on self-reports, which may overestimate the impact of the intervention. In this study, we used an objective, valid, highly sensitive, and reliable method [27], cotinine saliva testing, to distinguish smoking status.

### Limitations

This study has several limitations. Firstly, no more than 70% of participants responding at the third month follow-up might lead to the lack of power for detecting the hypothesized difference of 15%, although the high default rate was always challenging in conducting intervention research among migrants [47]. Besides, some participants declined to be followed up due to concerns about delaying their work and reducing their wages. As a remedy, we employed an intention-to-treat (ITT) approach and used multiple imputations by chained equation (MICE) analysis that would correct for bias in the intervention effect, under the assumption that data were missing at random given the observed covariates [48].

Secondly, with the intensive workload and lack of drinking, some participants’ mouths were too dry to offer sufficient saliva samples (at least 2 ML), which might threaten the tests of salivary cotinine concentrate. As a solution, we diluted these saliva samples with distilled water to testing volume and calculated the cotinine concentration taking the dilution ratio into account.

Finally, this was a small-scale feasibility study that demonstrated the initial effectiveness of the intervention, and we could not measure the sustained effect of the intervention. However, this study will inform the design and development of a larger-scale intervention of reducing smoking in the labor-intensive workplaces. In addition, the WHO 5A–based comprehensive intervention package piloted through this study may be replicable to the workplaces in another similar developing country context after some adaptation to local needs.

## Conclusions

The study demonstrated that a comprehensive smoking cessation intervention program based on the 5As was effective in improving smoking-related knowledge and attitude among migrant workers, though less effective in reducing the smoking rate. Future interventions should improve the migrant workers’ adherence to and participation in the 5A group counseling, and prioritize the intervention for migrant workers lacking in confidence in smoking cessation, with physiological illness, and with frequent migration.

## Additional files

Additional file 1: Study Protocol of this intervention. (PDF 210 kb)

Additional file 2: TREND Statement Checklist of this intervention. (PDF 509 kb)

## Abbreviations

CI, ITC: International tobacco control
DID: Difference-in-differences approach
EP: Eppendorf
GEE: Generalized estimating equations
ITT: Intention-to-treat
LC-MS/MS: Liquid chromatography-tandem mass spectrometry
MI: Multiple imputations
MICE: Multiple imputations by chained equation
OR: Odds ratio
WHO: World Health Organization

## References

[1] World Health Organization Global Health Risks: Mortality and burden of disease attributable to selected major risks. Geneva, Switzerland: World Health Organization, 2010.

[2] World Health Organization (2010). Global adult tobacco survey (GATS): China 2010 country report.

[3] Li S, Ma C, Xi B (2016). Tobacco control in China: still a long way to go. Lancet.

[4] Fong GT, Sansone G, Yan M, Craig L, Quah ACK, Jiang Y (2015). Evaluation of smoke-free policies in seven cities in China: longitudinal findings from the ITC China project (2007–2012), 2007-2012. Tob Control.

[5] World Health Organization Smoke-free Policies in China: evidence of effectiveness and implications for action. Geneva, Switzerland: World Health Organization, 2015.

[6] National Health and Family Planning Commission of the PRC. Report on China's Migrant Population Development (2015). Beijing: China Population Publishing House; 2015.

[7] Huang Z, Wang L, Zhang M, Deng Q, Wang Z, Zhao Y (2014). Smoking behavior among the Chinese employed floating population aged 18-59 in 2012. Zhonghua Liu Xing Bing Xue Za Zhi.

[8] Mou J, Fellmeth G, Griffiths S, Dawes M, Cheng J (2013). Tobacco smoking among migrant factory workers in Shenzhen, China. Nicotine Tob Res.

[9] Liu Y, Song H, Wang T, Yang H, Shen Y, Dai W (2015). Determinants of tobacco smoking among rural-to-urban migrant workers: a cross-sectional survey in shanghai. BMC Public Health.

[10] Arsenijevic J, Groot W. Lifestyle differences between older migrants and non-migrants in 14 European countries using propensity score matching method. Int J Public Health. 2017; 10.1007/s00038-017-1010-5. 10.1007/s00038-017-1010-5 PMC5978913 28707008

[11] Ali AK, Mohammed A, Thomas AA, Paul S, Shahul M, Kasim K (2017). Tobacco abuse and associated oral lesions among interstate migrant construction workers. J Contemp Dent Pract.

[12] Xie S (2017). The migration, mental stress, and tobacco use of internal migrants in China: the moderating effect of the social context of the host society. Subst Use Misuse.

[13] Thun, M. J., Carter, B. D., Feskanich, D., Freedman, N. D., Prentice, R., Lopez, A. D., Gapstur, S. M. (2013). 50-year trends in smoking-related mortality in the United States. N Engl J Med*,* 368(4), 351–364. Retrieved from 10.1056/NEJMsa1211127. 10.1056/NEJMsa1211127 PMC3632080 23343064

[14] Parrott, S., Godfrey, C., & Raw, M. (2000). Costs of employee smoking in the workplace in Scotland. Tob Control, 9(2), 187–192. Retrieved from 10.1136/tc.9.2.187. 10.1136/tc.9.2.187 PMC1748323 10841855

[15] Han YW, Mohammad M, Liew SM (2014). Effectiveness of a brief physician counselling session on improving smoking behaviour in the workplace. Asian Pac J Cancer Prev.

[16] Mallin R (2002). Smoking cessation: integration of behavioral and drug therapies. Am Fam Physician.

[17] Kim A, Kamyab K, Zhu J, Volpp K (2011). Why are financial incentives not effective at influencing some smokers to quit? Results of a process evaluation of a worksite trial assessing the efficacy of financial incentives for smoking cessation. J Occup Environ Med.

[18] Tanaka H, Yamato H, Tanaka T, Kadowaki T, Okamura T, Nakamura M (2006). Effectiveness of a low-intensity intra-worksite intervention on smoking cessation in Japanese employees: a three-year intervention trial. J Occup Health.

[19] Cahill K, Lancaster T (2014). Workplace interventions for smoking cessation. Cochrane Database Syst Rev.

[20] Fiore MC, Jaén CR, Baker TB, Bailey WC, Benowitz NL, Curry SJ, et al. Treating tobacco use and dependence: 2008 update. Clinical practice guideline*.* Rockville, MD: Department of Health and Human Services, 2008.

[21] World Health Organization, Toolkit for delivering the 5A's and 5R's brief tobacco interventions in primary care. Geneva, Switzerland: World Health Organization, 2014.

[22] Alomari MA, Khader YS, Dauod AS, Jordan TR, Khuder S (2013). Smoking cessation counseling practices of family physicians in Jordan. J Natl Med Assoc.

[23] Simmons VN, Litvin EB, Unrod M, Brandon TH (2011). Oncology healthcare providers’ implementation of the 5 A’s model of brief intervention for smoking cessation: patients’ perceptions. Patient Educ Couns.

[24] Liao J, Winickoff JP, Nong G, Huang K, Yang L, Zhang Z (2016). Are Chinese pediatricians missing the opportunity to help parents quit smoking?. BMC Pediatr.

[25] World Health Organization, The bill China cannot afford. The health, economic, and social costs of China’s tobacco epidemic. Geneva, Switzerland: World Health Organization, 2017.

[26] Statista. Distribution of WeChat users in China as of January 2015, by age*.* http://www.statista.com/statistics/387658/wechat-china-user-age/. (Accessed 21 June 2015).

[27] SRNT Subcommittee on Biochemical Verification (2002). Biochemical verification of tobacco use and cessation. Nicotine Tob Res.

[28] International Clinical Trials Registry Platform (ICTRP).Geneva: World Health Organization; 2014. (http://www.who.int/ictrp/en/).

[29] Vickers AJ, Altman DG (2013). Statistics notes: missing outcomes in randomized trials. BMJ.

[30] World Health Organization WHO Framework Convention on Tobacco Control. Geneva, Switzerland: World Health Organization, 2003.

[31] Spires M, Rutkow L, Feldhaus I, Cohen JE (2014). The World Health Organization's MPOWER framework and international human rights treaties: an opportunity to promote global tobacco control. Public Health.

[32] Wipfli HL, Samet J (2015). Framing progress in global tobacco control to inform action on non-communicable diseases. Health Aff (Millwood).

[33] Mohammadnezhad M, Tsourtos G, Wilson C, Ratcliffe J, Ward P (2015). “I have never experienced any problem with my health. So far, it hasn't been harmful”: older Greek-Australian smokers' views on smoking: a qualitative study. BMC Public Health.

[34] Intarut N, Chongsuvivatwong V, McNeil E (2016). Effects of a school-based intervention program on attitude and knowledge of household members towards a smoke-free home: a cluster controlled trial. Asian Pac J Cancer Prev.

[35] Tahlil T, Woodman RJ, Coveney J, Ward PR (2015). Six-months follow-up of a cluster randomized trial of school-based smoking prevention education programs in Aceh, Indonesia. BMC Public Health.

[36] Wen X, Chen W, Gans KM, Colby SM, Lu C, Liang C (2010). Two-year effects of a school-based prevention programme on adolescent cigarette smoking in Guangzhou, China: a cluster randomized trial. Int J Epidemiol.

[37] Rollins C, Glass NE, Perrin NA, Billhardt KA, Clough A, Barnes J,et al. Housing instability is as strong a predictor of poor health outcomes as level of danger in an abusive relationship: findings from the SHARE study. J Interpers Violence 2012; 27: 623–43. 10.1177/0886260511423241 21987519

[38] Okechukwu CA, Krieger N, Sorensen G, Li Y, Barbeau EM (2009). Effectiveness of an apprenticeship site-based smoking cessation intervention for unionized building trade workers. Cancer Causes Control.

[39] Bergström G, Björklund C, Fried I, Lisspers J, Nathell L, Hermansson U (2008). A comprehensive workplace intervention and its outcome with regard to lifestyle, health and sick leave: the AHA study. Work.

[40] Kadowaki T, Okamura T, Funakoshi T, Okayama A, Kanda H, Miyamatsu N (2004). Effectiveness of annual interventions for smoking cessation in an occupational setting in Japan. Environ Health Prev Med.

[41] Iliceto P, Fino E, Pasquariello S, D’ Angelo Di Paola ME, Enea D (2013). Predictors of success in smoking cessation among Italian adults motivated to quit. J Subst Abus Treat.

[42] Dorner T, Tröstl A, Womastek I, Groman E (2011). Predictors of short-term success in smoking cessation in relation to attendance at a smoking cessation program. Nicotine Tob Res.

[43] Yong HH, Hamann SL, Borland R, Fong GT, Omar M, project team ITC-SEA (2009). Adult smokers' perception of the role of religion and religious leadership on smoking and association with quitting: a comparison between Thai Buddhists and Malaysian Muslims. Soc Sci Med.

[44] Hagimoto A, Nakamura M, Morita T, Masui S, Oshima A (2009). Smoking cessation patterns and predictors of quitting smoking among the Japanese general population: a 1-year follow-up study. Addiction.

[45] Hellman R, Cummings KM, Haughey BP, Zielezny MA, O'Shea RM (1991). Predictors of attempting and succeeding at smoking cessation. Health Educ Res.

[46] Vangeli E, Stapleton J, Smit ES, Borland R, West R (2016). Predictors of attempts to stop smoking and their success in adult general population samples: a systematic review. Addiction.

[47] Spicer RS, Miller TR (2016). The evaluation of a workplace program to prevent substance abuse: challenges and findings. J Prim Prev.

[48] Zhang Z (2016). Multiple imputation with multivariate imputation by chained equation (MICE) package. Ann Transl Med.

