# Supplementary material for: Evaluation of the effectiveness of a WHO-5A model based comprehensive tobacco control program among migrant workers in Guangdong, China: a pilot study
Source: BMC Public Health. 2018 Feb 27;18:296. doi: 10.1186/s12889-018-5182-6 (PMC6389256; doi:10.1186/s12889-018-5182-6)
Supplement: Supplementary file 1 — Study Protocol of this intervention. (PDF 210 kb) [file 12889_2018_5182_MOESM1_ESM.pdf]

Study protocol: Development of a Tobacco Control Trial among Migrant Workers in Guangzhou, China

Background

It have been widely accepted that cigarette smoking is of tremendous hazards to health, so as the exposure to Secondhand Smoke (SHS). Cigarette smoking increases the incidence of maternal cardiovascular and pulmonary events and other comorbid conditions among pregnant smokers[1]. Women make up 80% of the total disease burden of SHS[2]. Kinds of respiratory symptoms were more frequently observed among adults exposed to SHS[3,4].

China signed WHO Framework Convention on Tobacco Control (FCTC) in 2003. However, there is still a significant gap between the current state of tobacco control and the requirements of the FCTC, mainly due to the state tobacco monopoly in China[13].

As reported by the Global Adult Tobacco Survey (GATS)[6], in 2010, an estimated 28.1% of adults in China (52.9% of men and 2.4% of women) currently smoked. As reported by nonsmokers, 72.4% had been exposed to secondhand smoke and 38.0% were exposed to secondhand smoke on a daily basis[7]. The direct medical cost of smoking-related morbidity and mortality was estimated at 22.9 billion Chinese Yuan[5], and keeps increasing at a staggering rate.

In China, internal rural-to-urban migrants make a large proportion of the whole population, accounting for about 0.245 billion (18 % of the total population) in 2013. The national data of smoking in migrant population is still lacking. Studies shows that in Beijing, the total smoking rate of migrant population was19.2% (48.7% for male and 2.1% for female)[8] and in another study was 51.7% (male) and 10.9% (females)[9]. One study in Zhejiang province found that 29.83% of 1086 migrant population have smoked, 54.09% of whom weremales[10]. A survey of five areas of Jiangxi province shown the total smoking rate was 32.8% (56.3% for male and 2.5% for female)[11]。Cui et al found the overall smoking prevalence among migrant workers was 64.9% (95% CI: 62.4-67.2%)[12]. These researches shown that the smoking rate was generally higher in migrant population than local residents.

Interventions targeting at migrant population on reducing smoking, including workplace interventions are still scare. Zhong[15], applied knowledge education to in workplaces, with smoking rate reduced from 42.9% to 28.9%. Gao[16], used a health promotion strategy in a factory of Shanghai, making the smoking rate decreased from 55.8% to 34.1%.

Tobacco control intervention for migrant population often involved the general factors like hazard awareness[17], diseases prevention, family pressure and financial pressure[18]. Research also shown that migratory lifestyle, such as social changes and stresses caused by rural to urban migration, was associated with smoking initiation[19]. The migrant span, migrant history, discrimination and social support all contribute to smoking[20-23]. However, it is unknown to what extent the comprehensive intervention that considers these factors can be effective in

reducing the smoking rate and SHS exposure for the migrants in the workplace settings in China.

Research questions

1. How can the WHO 5A's model be adapted into 5A's group consulting intervention package that fits migrant workers in China?

2. To what extent it is feasible and acceptable of delivering the intervention in the workplaces setting, and how the evidence generated from this study will inform the design of the main trial.

Methods/Design

This is a controlled before and after designed pilot trial to be conducted in manufacturing factories in Zhongshan City, China.

Study design

First, the package would be designed by tailoring the WHO 5A's model into 5A's group consulting intervention package through literature review, in-depth interview and focus group discussion. Then a pilot trial of the package will be carried out in 4 factories in Zhongshan industrial zone. The primary outcome of this pilot trial will be the feasibility and acceptability

of delivering the intervention and feasibility of assessing its outcomes. The secondary outcome includes information to inform the design of the future RCT, such as sample size, and facilitators and barriers of implementation in industrial settings.

Objective 1

To tailor the WHO 5A's model into 5A's group consulting intervention package that fits migrant workers.

Key activities

Based on literature review on articles related to migrant workers' smoking and SHS exposure, as well as the WHO 5A's model, we will conduct in-depth interview and focus group discussion to understand: 1) Which specific factors affecting migrant workers' smoking cessation should be considered in the intervention. 2) In which way the group consulting intervention should be applied in the industrial setting. 3) What specific content should be contained in the group consulting package to make the package effective?

Settings & Participants

In-depth interviews will conducted with 4-6 factory managers and 6-8 migrant workers. Four focus groups with 6-8 factory managers/workers/health educators in each group will be conducted in factories.

Data collection

Facilitator guided discussions (FDG) will be conducted by the research team. The FGD guideline will be constructed with the help of factory managers/workers/health educators, and will be pilot tested with two in-depth interviews and a focus group. All the FDGs will be

recorded.

Objective 2

To test the feasibility and acceptability of delivering the intervention, and provide information to the design of the main trial such as outcome suitability, number of clusters (factories) and size of each cluster (participants), effect size, intra-cluster correlation coefficient (ICC) , recruitment and attrition rates.

Key activities

We aim to recruit 8 factories (clusters) and all the migrant workers working in these factories from industrial zone of Guangzhou, China. The clusters will be randomized to the intervention and control group in a 1:1 ratio. Clusters allocated to the intervention arm will be offered the 5A's group consulting package. The clusters in the control arm will not be offered the package until the completion of the study. All the migrant workers who work in each factory and provide informed consent will be recruited. Factories will complete a factory survey of basic factory information, all participants will complete a questionnaire (about the status of tobacco exposure, knowledge and attitude of tobacco, and demographic information), and a non-smoking individual will provide a salvia sample which will be tested for cotinine. All these participant outcomes (questionnaire and salvia cotinine) will be measured before and after the 3-month intervention in both arms of the trial. In addition, a purposive sample of participants will be invited for interviews to investigate the facilitators and barriers for integrating 5A's group consulting package into workplace settings and how these can be enhanced or addressed at the end of this trial.

The 5A's group consulting package intervention includes:

1. Factsheets detailing key information on smoking, SHS.
2. Guidelines for group guiders on how to deliver the 5A’s group consulting (activities for different audiences: smokers, non-smokers exposed to SHS).
3. A leaflet that contains the key facts about smoking and SHS that can be disseminated to migrant workers after consulting.

Eligibility criteria

Inclusion criteria for cluster recruitment: 1. Factory should be labor intensive in manufacture industry. 2. Have at least 80 migrant workers that can participate in the trial. 3. The manager should be willing to adhere to the planed intervention. Exclusion criteria: 1. Have taken part in a similar tobacco control activity before.

We plan to recruit all the migrant workers working in each factory. We will include rural to urban migrants, no matter whether they smoke or not, who are willing to participate in the trial. We will exclude workers who do not smoke and have no SHS exposure at all.

Data collection

For objective 2, we have mainly outcomes as follows:

**Category**	**Variables**	**Methods**	**Population targeted**	**Outcomes**
**Primary outcome**	Feasibility	Questionnaire in intervention group and in-depth interview	Intervention deliver, migrant workers and factory manager	1.Satisfaction rate<br>2.Feasibility score
	Acceptability	Questionnaire in	Intervention deliver,	1.Satisfaction rate

		intervention group and in-depth interview	migrant workers and factory manager	2.Intent to use rate
**Secondary outcome**	Salivary cotinine	Salivary cotinine test conducted before and after the intervention	Non-smoking migrant workers	1.Salivary cotinine concentration
	Smoking status	Questionnaire survey conducted before and after the intervention	Smoking migrant workers	1.Smoking quit rate
	Knowledge, attitude	Questionnaire survey conducted before and after the intervention	Migrant workers	1.Knowledge change<br>2.Attitude change
	Attrition rate, intraclass correlation coefficient(ICC)	The whole research progress	Migrant workers	1.Attrition rate<br>2.ICC

For each factory in the study the following information will be recorded at the start of the study: Factory scale, types of factory products, demographic composition.

At baseline, each migrant participant will complete a questionnaire encompasses four main dimensions: (1) demographic characters; (2) migrant characters; (3) smoking behaviors and practices; (4) smoking related knowledge and attitudes. The same dimensions will be covered in the follow up survey three months after the baseline survey.

Cotinine is a metabolite of nicotine, and can be most sensitively and specifically measured by salivary cotinine samples, which have a half-life period of 12 to 18 hours and thus can reflect the degree of tobacco exposure[25,26]. We will thus collect a saliva sample to measure salivary cotinine levels at baseline and a second saliva sample from the same participants three months after intervention start.

We will conduct in-depth interviews with twenty participants, five package delivers and five factory managers at baseline, and at program completion (3 months post baseline). All the interview contents will be recorded.

Intervention fidelity

To maintain the quality of the intervention delivery and track fidelity to the intervention, we will use the following approaches. Firstly, the team leaders will be asked to submit an implementation form every week to research team to report the adherence to protocol, barriers to the implementation of the intervention and their suggestions. PI, Co-PI and research team of this project will look over these forms and provide feedback to all the team leaders to help them to conduct the interventions better. Secondly, migrant workers will also self-report the adherence to the intervention, the barrier to quit smoking and the satisfaction to the team leaders' work by instant messaging to the research team directly. In addition, at the end of each month, the research team will go to the intervention factories to talk with the team leaders about intervention progress and offer some professional resolutions. As for those

unqualified team leaders, they will be re-trained or replaced.

Data management

Data will be collected at baseline, and at program completion (3 months post baseline).

To avoid missing and logic mistakes in the questionnaires, all completed questionnaires will be checked by the interviewers when submitted, and the unqualified items in the questionnaires will be asked to modify by the migrant workers. All participants will provide at least 2mL saliva samples for salivary cotinine concentrate test. We will require all participants to refrain from eating, drinking, or smoking at least one hour before sample collection. We will collect salivary samples on the day of transport, and samples will be labeled and stored in EP tubes (Eppendorf Tubes) at -20℃ until laboratory analysis. Samples will be analyzed using liquid chromatography-tandem mass spectrometry (LC/MS/MS) method. All experiments will be duplicated. The laboratory technicians will be blinded to participants' smoking status.

For those are lost to follow-up, the reasons of dropping out will be recorded. Before data can be analyzed, it will be processed, including correcting data-inputting errors by using double data entry method and range and logic checks. All personal information about the enrolled participants will not be shared with any third party during and after the trial to protect confidentiality. Questionnaires will be well stored in a locked room. Saliva will be destroyed after detection. Data will be stored in a secure database with password encrypted.

Data analysis

Data from baseline and follow-up will be analyzed using descriptive statistics (means, standard deviations, frequencies, etc.). The level of significance will be set at 0.05. IBM SPSS 20.0 and STATA 12 will be employed for analysis. Data will be analyzed to evaluate program fidelity, adaptations, effectiveness, factors that facilitated and challenged program delivery, and recommendations for improvement. Analyses will follow the intention-to-treat (ITT) principle; multiple imputation chained equation (MICE) method will be used to address missing or incomplete data. Difference-in-difference (D-in-D) analyses will be usedto evaluate the effectiveness of the intervention. Logistic regressions and multiple linear regressions will be respectively employed to explore factors associated with the change of the outcome variables over time in the intervention arm, and results were represented as odds ratios (OR) with 95% confidence intervals (95% CI).

Acknowledgements

We would like to thank all migrant workers participated in this study and the support from the Health and Family Planning Commission of Zhongshan City and the Health and Family Planning Bureau of Xiaolan Town, Zhongshan. We sincerely acknowledge JiazhiZeng, Yong Xu, Liwei Deng, Tongyang Li and AoLuo from Sun Yat-sen University, and Simin Deng and Jia Yin from the Chinese University of Hong Kong for their assistance with the implementation of the intervention and/or data collection.

References:

1 Roelands, J., Jamison, M. G., Lyerly, A. D., & James, A. H. (2009).Consequences of smoking during pregnancy on maternal health. Journal of Women's Health, 18(6), 867-872.
2 Gan Q, Smith KR, Hammond SK et al. Disease burden of adult lung cancer and ischaemic heart disease from passive tobacco smoking in China. Tob. Control 2007; 16: 417–22.
3 Dong GH, Wang D, Yang ZH et al. Gender-specific differences in effects of prenatal and postnatal environmental tobacco smoke exposure on respiratory symptoms in 23,474 children with and without allergic predisposition: results from 25 districts of northeast China. Int. J. Environ. Health Res. 2011; 21: 1–16.
4 Dong GH, Ren WH, Wang D et al. Exposure to secondhand tobacco smoke enhances respiratory symptoms and responses to animals in 8,819 children in kindergarten: results from 25 districts in northeast China. Respiration 2010; 81: 179–85.
5 Chen MH. Economic concerns hamper tobacco control in China. Lancet 2007; 370: 729–30.
6 Li, Qiang, Jason Hsia, and Gonghuan Yang."Prevalence of smoking in China in 2010." New England Journal of Medicine 364.25 (2011): 2469-2470.
7 Yang, G. H. "Global Adult Tobacco Survey (GATS) China 2010 Country Report." China: Chinese Three Gorge Press (2011)
8 YU Zhen,QIAOLei,WANGXu-hong,et al. Investigation on health-threatening behaviors among the immigrants living in an urban district of Beijing. Chinese Journal of Prevention and Control of Chronic Diseases, 2010,05:448-451.
9 Chen, Xinguang, et al. "Cigarette smoking among rural-to-urban migrants in Beijing, China." Preventive medicine 39.4 (2004): 666-673.
10 ZHU Zhou-na,ZHANG Ming-jun,ZHONG Su-ya. Investigation of health related factors of migrant workers gathering areas.Chinese General Practice, 2013,23:2140-2142.
11 HE Jun-rong, ZHU Li-ping, WANG Rui-ping, et al. Analysis on the smoking situations and tobacco costs among floating population in Jiangxi Province. Chinese Journal of Disease Control & Prevention, 2014,01:58-62.
12 Cui, X., et al., Work stress, life stress, and smoking among rural-urban migrant workers in China. BMC Public Health, 2012. 12: p. 979.
13 Lv, Jun, et al. "Implementation of the WHO framework convention on tobacco control in mainland China." Tobacco control (2011): tc-2010.
14 China tobacco control plan (2012-2015). http://www.tobacco.gov.cn/html/49/4254349_n.html
15 ZhongFei. Research of smoking related KAP and workplace intervention among workers. Sichuan University, 2006.
16 GaoJianing. Tobacco control in workplace with ecological model of health promotion.Fudan University, 2010.
17 Jiang Bin, Wang Wenzhi, Wu Shengping, Hong Zhen,et al. Evaluation on the effects of community-based anti-smoking intervention. Journal of Hygiene Research, 2003(05): 477-479.
18 Hesketh, T., et al., Smoking, cessation and expenditure in low income Chinese: cross sectional survey. BMC Public Health, 2007. 7: p. 29.
19 Yang T, Wu J, Rockett IR et al. Smoking patterns among Chinese rural-urban migrant workers. Public Health 2009; 123: 743–9.
20 Cao X, Yu L, Zhou H, Wu F, Ma X. Smoking and its impact factors among migrant workers in Chengdu. Modern Preventive Medicine, 2010,15:2860-2862.
21 Chen, X., et al., Cigarette smoking among rural-to-urban migrants in Beijing, China.Prev Med, 2004. 39(4): p. 666-73.
22 Mou, J., et al., Tobacco smoking among migrant factory workers in Shenzhen, China.Nicotine Tob Res, 2013. 15(1): p. 69-76.
23 Cui, X., et al., Work stress, life stress, and smoking among rural-urban migrant workers in

China. BMC Public Health, 2012. 12: p. 979.
24 WHO: Toolkit for delivering the 5A’s and 5R’s brief tobacco interventions in primary care. http://www.who.int/tobacco/publications/smoking_cessation/9789241506953/en/
25 Russell, T. V., Crawford, M. A., &Woodby, L. L. (2004). Measurements for active cigarette smoke exposure in prevalence and cessation studies: why simply asking pregnant women isn't enough. Nicotine & Tobacco Research, 6(Suppl 2), S141-S151.
26 Wall, M. A., Johnson, J. E. A. N., Jacob, P. E. Y. T. O. N., &Benowitz, N. L. (1988). Cotinine in the serum, saliva, and urine of nonsmokers, passive smokers, and active smokers.American journal of public health, 78(6), 699-701.
